# NK Cells in the Treatment of Hematological Malignancies

**DOI:** 10.3390/jcm8101557

**Published:** 2019-09-27

**Authors:** Ana P Gonzalez-Rodriguez, Mónica Villa-Álvarez, Christian Sordo-Bahamonde, Seila Lorenzo-Herrero, Segundo Gonzalez

**Affiliations:** 1 Department of Hematology, Hospital Universitario Central de Asturias (HUCA), 33011 Oviedo, Spain; anapilargonzalez@gmail.com; 2 Instituto Universitario de Oncología del Principado de Asturias, IUOPA, 33006 Oviedo, Spain; movialnk@gmail.com (M.V.-Á.); christiansbl87@gmail.com (C.S.-B.); seilalorenzoherrero@gmail.com (S.L.-H.); 3 Instituto de Investigación Biosanitaria del Principado de Asturias (IISPA), 33011 Oviedo, Spain; 4 Department of Functional Biology, Immunology, University of Oviedo, 33006 Oviedo, Spain; 5 EntreChem S.L., 33011 Oviedo, Spain

**Keywords:** NK cells, cancer, immunotherapy, hematopoietic stem cell transplantation, checkpoint, CAR-NK

## Abstract

Natural killer (NK) cells have the innate ability to kill cancer cells, however, tumor cells may acquire the capability of evading the immune response, thereby leading to malignancies. Restoring or potentiation of this natural antitumor activity of NK cells has become a relevant therapeutic approach in cancer and, particularly, in hematological cancers. The use of tumor-specific antibodies that promote antibody-dependent cell-mediated cytotoxicity (ADCC) through the ligation of CD16 receptor on NK cells has become standard for many hematologic malignancies. Hematopoietic stem cell transplantation is another key therapeutic strategy that harnesses the alloreactivity of NK cells against cancer cells. This strategy may be refined by adoptive transfer of NK cells that may be previously expanded, activated, or redirected (chimeric antigen receptor (CAR)-NK cells) against cancer cells. The antitumor activity of NK cells can also be boosted by cytokines or immunostimulatory drugs such as lenalidomide or pomalidomide. Finally, targeting immunosubversive mechanisms developed by hematological cancers and, in particular, using antibodies that block NK cell inhibitory receptors and checkpoint proteins are novel promising therapeutic approaches in these malignant diseases.

## 1. Introduction

**Figure 1:**
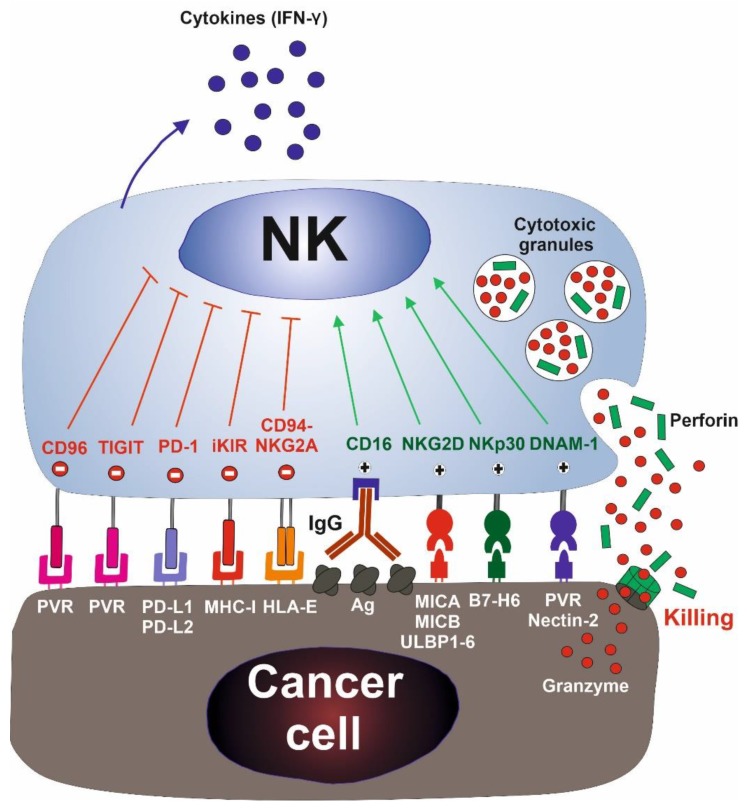
Natural killer (NK) cell activation: The activation of NK cells is mediated by a balance of signals provided by a network of activating and inhibitory receptors. Inhibitory receptors (depicted in red) recognize surface self-proteins normally expressed by all healthy nucleated cells. The loss of their expression, frequently caused by viral infection or cellular transformation, leads to NK cell activation (“missing self” recognition). Activating receptors (depicted in green) recognize ligands that are induced on virus-infected and malignant cells. Activated NK cells induce the apoptosis of tumor cells by the exocytosis of cytotoxic granules containing perforin and granzymes, and secrete cytokines, such as IFN-γ. Major inhibitory and activating receptors on NK cells and their cognate ligands on targets are depicted. IFN-γ, interferon-γ; TIGIT, T cell Ig and ITIM domain; PD-1, programmed death-1; iKIR, inhibitory killer cell immunoglobulin-like receptor; NKG2A, natural killer group 2A; NKG2D, natural killer group 2D; NKp30, natural killer P30; DNAM-1, DNAX accessory molecule 1; PVR, polivirus receptor; PD-L1 and 2, programmed death-ligand 1 and 2; MHC-I, MHC class I; HLA-E, human leucocyte antigen E; Ag, antigen; MICA, MHC class I polypeptide-related sequence A; MICB, MHC class I polypeptide-related sequence B; ULBP1-6, UL16 binding proteins 1–6.

Natural killer (NK) cells are cytotoxic immune cells that have been functionally identified by their “natural“ ability to kill tumor cell lines in vitro [1]. These cells constitute 5% to 15% of mononuclear cells in blood and lymphoid organs. They play a central role in the immune response against viral infection and cancer. Contrasting to T lymphocytes, NK cells do not recognize antigens, instead, they distinguish cancer cells from their healthy counterparts through the function of an array of activating and inhibitory receptors that recognize self-proteins expressed on the cellular surface (Figure 1). Inhibitory receptors recognize surface self-proteins. The loss of their surface expression, frequently caused by viral infection or cellular transformation, leads to NK cell activation (“missing self” recognition) [2]. Inhibitory killer cell immunoglobulin-like receptors (KIRs) and the heterodimer CD94-natural killer group 2A (NKG2A) are major inhibitory receptors that recognize self-human leucocyte antigen class I molecules (HLA-I) [3]. NK cells may be further regulated by inhibitory receptors best known as checkpoint proteins, such as programmed death-1 (PD-1), which may play an important role in modulating NK cell activity in cancer [4].

Activating receptors bind to their corresponding ligands restrictedly expressed on infected and malignant cells. These receptors include natural killer group 2D (NKG2D), DNAX accessory molecule 1 (DNAM-1), and the natural cytotoxicity receptors natural killer P30, 44, and 46-related protein (NKp30, NKp44, and NKp46) [3,5,6]. NKG2D is a key activating receptor that binds to MHC class I polypeptide-related sequence A (MICA), MHC class I polypeptide-related sequence B (MICB), and UL16 binding proteins 1–6 (ULBP1-6) molecules [7]. NKG2D ligands (NKG2DL) are restrictedly expressed on normal cells to avoid autoimmunity, but they are upregulated in transformed and virus-infected cells acting as a stress signal capable of activating NK cells [8,9]. Additionally, NK cells may kill tumor cells that have been bound by specific IgG_1_ and IgG_3_ antibodies through CD16 receptor (also named FcγRIII), a process known as antibody-dependent cell-mediated cytotoxicity (ADCC). This is a relevant mechanism of action of some therapeutic monoclonal antibodies (mAbs) used in the treatment of cancer (see below) [3,6].

After activation, NK cells induce the apoptosis of tumor cells by the exocytosis of cytotoxic granules containing perforin and granzymes [5]. Additionally, NK cells may also kill target cells via the extrinsic pathways of apoptosis (TNF-related apoptosis-inducing ligand (TRAIL) or Fas) and secrete cytokines, such as interferon-γ (IFN-γ) that favor adaptive and innate immune antitumor responses.

## 2. Evidence of Immune Surveillance of Hematological Cancers by NK Cells

The role of NK cells in the elimination of cancer has been controversial for more than 30 years [10]. A major role of NK cells in the control of tumor growth and metastasis has been well established in animal models [10,11], but their relevance in the immune surveillance of human tumors is still an open question. Nevertheless, the observations in experimental models significantly correlate with a wide range of clinical data clearly suggesting that NK cells are highly relevant in the immune response to hematological malignancies and metastases [11,12,13] (Table 1).

Selective NK cell human deficiencies are extremely rare [14], however, they are associated with the development of lymphoproliferative disorders [15]. Germline mutations of perforin 1 gene (*PRF1*) are frequently found in patients with childhood anaplastic large cell lymphoma (ALCL) and acute lymphoblastic leukemia (ALL) [16,17]. Likewise, a significant proportion of patients with lymphoma harbors mutations that inactivates perforin and Fas ligand (*FASLG*) genes, which are associated with absent NK cell activity [18]. Accordingly, individuals with higher levels of natural cytotoxic activity of blood lymphocytes showed a decreased risk of cancer in an 11-year follow-up cohort study [19]. Of note, it remains to be elucidated whether the increase of malignancies experienced by these patients is dependent on their higher susceptibility to viral infection, particularly to Epstein–Barr viral infection, owing to their defective NK cell-dependent antiviral immunity.

The number and activity of NK cells have been associated with prognosis in several hematological cancers. Thus, the presence of NK cells in the bone marrow of patients with ALL at diagnosis is associated with better responses to treatment and higher chances of remission [20]. Likewise, a prevalence of NK cells with a strong effector phenotype at diagnosis is associated with the control of the disease after chemotherapy treatment [21]. The number and function of NK cells have also been associated with the severity and prognosis of pediatric non-Hodgkin’s lymphomas (NHL), chronic lymphocytic leukemia (CLL) [22,23,24], and diffuse large B-cell lymphoma (DLBCL) [25]. In addition, secretion of IFN-γ by NK cells is also a positive prognostic marker in chronic myeloid leukemia (CML) [12], while reduced NK cell function is associated with high-risk myelodysplastic syndrome (MDS) [26]. Although these data suggest a role of NK cells in the control of these malignancies, it remains to be fully established whether this increase in the number or activity of NK cells is the consequence of the cancer development or it reflects a significant antitumor immune response. Furthermore, a correlation between NK cells and outcome is controversial in the context of some malignancies. Thus, in multiple myeloma (MM), decreased NK cell activity has been positively associated with advanced clinical stage and worse survival [27], whereas higher numbers of NK cells correlated with high-risk disease and poorer prognosis in a different study [28]. Nevertheless, this study also showed that patients with higher values of CD57^+^CD8^-^ cells, which included a high number of NK cells, have better outcome, hence suggesting a different NK cell subpopulation distribution between both studies. Alternatively, these apparently contradictory results may be due to the existence of different levels of NK cell activity [29]. Higher NK cell numbers have also been associated with lower survival in cutaneous T cell lymphoma [30]. Although, the cause of this association is unknown, it suggests that the activity of NK cells may be impaired.

Despite the well-established antitumor role of the immune system, particularly in the initial stages of cancer, advanced tumors generally develop a plethora of evasion mechanisms counteracting NK cell-mediated antitumor response [31]. In agreement, a progressive reduction of the NK cell number and functionality is frequently observed in patients with cancer, which leads to a worse prognosis [32]. Phenotypically, NK cells acquire an inhibitory phenotype, characterized by an increased expression of inhibitory receptors and other immunosuppressive proteins, as well as lower levels of activating receptors and their ligands on tumor cells [32].

**Table 1:** Clinical implications and role of NK cells in the pathogenesis of hematological malignancies. ALCL, anaplastic large cell lymphoma; ALL, acute lymphoblastic leukemia; CLL, chronic lymphocytic leukemia; AML, acute myeloid leukemia; MDS, myelodysplastic syndrome; CML, chronic myeloid leukemia; HL, Hodgkin’s lymphoma; NHL, non-Hodgkin’s lymphoma; DLBCL, diffuse large B-cell lymphoma; MM, multiple myeloma; NK, natural killer; *PRF1,* perforin 1 gene; NKG2D, natural killer group 2D; NKG2DL, NKG2D ligands; NKp30, 44, 46, natural killer P30, 44, 46; TIM-3, T cell immunoglobulin domain, mucin domain; FASLG, Fas ligand gene; ULBP1, UL16 binding proteins 1; NKG2A, natural killer group 2A.

Malignancy	NK Cell Phenotype and Function	Clinical Significance	References
**ALCL, ALL**	Perforin 1 gene *(PRF1)* mutations	Predisposition to disease	[16,17]
**ALL**	NK cells in bone marrow at diagnosis	Prognostic factor in children	[20]
	Strong NK cell effector phenotype	Correlation with minimal residual disease	[21]
**CLL**	NK cell number	Correlation with disease stage and prognosis	[22,23,24]
	Soluble NKG2DL production	Correlation with poor prognosis	[33,34]
	NKp30 downregulation, TIM-3 upregulation	Correlation with poor prognosis	[35]
**AML**	Soluble ULBP1 production	Correlation with poor prognosis	[36]
	NKp30, NKp44, NKp46 downregulation	Correlation with poor prognosis	[37]
	CD94/NKG2A upregulation	Reduced effectiveness of chemotherapy	[38]
**MDS**	Reduced NK cell function and NKG2D downregulation	Association with high-risk disease	[26]
**CML**	NKG2D downregulation	Imatinib restored NKG2D expression	[39]
**HL, NHL**	*PRF1* and *FASLG* mutations. Absent NK cell activity	Predisposition to disease	[18]
**DLBCL**	Reduced NK cell numbers	Correlation with poor prognosis	[25]
**Burkitt lymphoma**	Reduced cytotoxicity and NKp46, NKp30 and CD160 expression	Correlation with poor prognosis	[40]
**T cell lymphoma**	Higher NK cell numbers	Correlation with poor prognosis	[30]
**MM**	NK cell number and function	Contradictory results between studies	[27,28]
	Soluble MICA production	Correlation with poor prognosis	[41]
	Soluble CD16 production	Association with disease stage	[42]

NKG2D is a paradigm of NK cell activating receptors and its role in hematological cancers has been extensively analyzed. A crucial evasion mechanism of the NKG2D-mediated immune response is the production of soluble NKG2DL by cleaving them from the membrane of malignant cells. Shedding of soluble MICA depends on its interaction with the chaperone ERp5 on the surface of tumor cells [43], which allows its proteolytic cleavage by proteases [44,45]. Hence, ERp5 was associated with soluble MICA shedding in CLL [46], MM [47], and Hodgkin’s lymphoma (HL) [48]. The shedding of soluble NKG2DL induces the internalization of surface NKG2D receptor impairing NK cell activity and favoring immune evasion. In agreement, increased levels of soluble NKG2DL correlate with more aggressive clinical course and poor survival in different hematological malignancies and solid tumors [49]. Thus, soluble ULBP2 is an independent indicator of poor prognosis in CLL [33] and MM [50], while soluble MICA is an independent prognostic factor in MM [41]. Similarly, decreased NKG2D expression and increased shedding of soluble NKG2DL are associated with advanced stage, progressive disease, and poor prognosis in CLL [33,34]. In CML, the BCR-ABL fusion protein downregulates the expression of NKG2D on NK cells, through the chronic exposure to increased levels of NKG2DL. However, treatment with the tyrosine kinase inhibitor imatinib restored the expression of NKG2D in these patients [39]. Similarly, the expression of some NKG2DL, such as ULBP1, correlates with prognosis and survival after chemotherapy treatment in AML [36].

Other key activating receptors, such as natural cytotoxicity receptors or NCRs (NKp30, NKp44, and NKp46) are downregulated in acute myeloid leukemia (AML) correlating with poor prognosis and lower survival rates [37]. NK cells with reduced cytotoxicity and lower expression of NKp46, NKp30, and CD160 have also been observed in Burkitt lymphoma and CLL [40]; while increased expression of the T-cell immunoglobulin and mucin domain-3 (TIM-3) inhibitory receptor on NK cells correlates with a poor prognosis in CLL [35]. Besides, serum levels of soluble CD16 are linked to disease stage in MM patients [42]. Contrarily, in patients with AML, the upregulation of the inhibitory receptor CD94/NKG2A on NK cells may be responsible for their diminished effector function, reducing the possibility of complete remission after chemotherapy [38]. These accumulating data clearly support a role for NK cells in the immune surveillance of hematological malignancies, nevertheless, the relative contribution of NK cells compared to other immune cells needs to be fully established, since defective NKT o T cell function may also contribute to the phenotype observed.

The capability of NK cells to eliminate leukemia cells has been further established in hematopoietic stem cell transplantation (HSCT). NK cells directly kill cancer cells and reduce the incidence of graft-versus host disease (GvHD) [51]. In HSCT, a rapid recovery of NK cells and a higher NK cell count in the graft have been associated with better outcome [52,53]. In the haploidentical HSCT, NK cell-mediated alloreactivity may remarkably increase the survival of patients with AML who are devoid of HLA-I molecules that match donor-inhibitory KIR receptor (see below) [54,55]. Significantly, allogeneic NK cells preferentially kill poor prognosis leukemia cells (*TP53* mutated) that are resistant to classical chemotherapeutic drugs [56]. Furthermore, the immunosuppressive profile of NK cells frequently observed in advanced cancers may significantly reduce the efficacy of HSCT [57,58] and other NK cell-based therapies [59,60]. Impaired NK cell-cytotoxicity also interferes with the response to chemotherapy with azacitidine (AZA) and reduces the survival of patients with AML [61], suggesting that NK cell function may also play a significant role in the response to more conventional chemotherapeutic agents.

To conclude, due to the paucity of cases of selective NK cell deficiency [14], the role of NK cells in the surveillance of human cancers remains an open question. Nevertheless, robust experimental data [10,11], which correlate with wide clinical data described above, clearly support that NK cells play a role in the control of the development and progression of hematological malignancies. These observations also indicate that advanced cancers develop multiple mechanisms of immune evasion impairing the efficacy of their antitumor immune response [31,32]. Consequently, the potentiation or restoration of this innate antitumor activity of NK cells constitutes potential strategies for the immunotherapy of hematological cancers [62].

## 3. Anticancer Therapies Involving NK Cell Modulation

**Figure 2:**
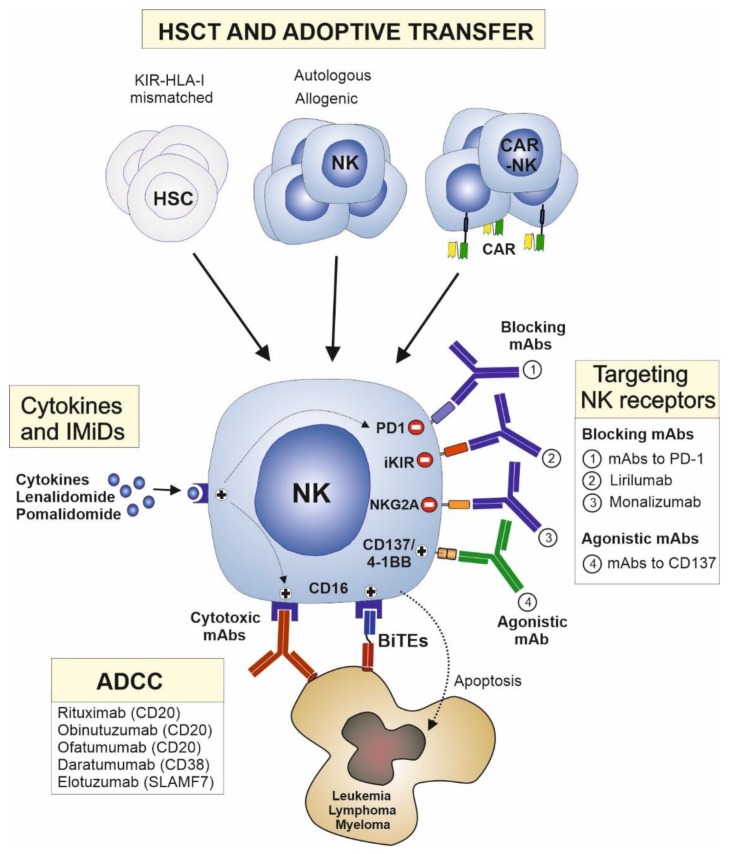
Therapeutic approaches involving natural killer (NK) cells to treat hematological cancers. Cytotoxic mAbs that engage CD16 receptors on NK cells and induce antibody-dependent cell-mediated cytotoxicity (ADCC) are the most widely used NK cell-based therapies in hematological cancers. The so-called bispecific antibodies (BITE) may improve ADCC activity by redirecting NK cells to tumor cells. NK cells, and particularly allogenic NK cells that are devoid of inhibitory KIRs for donor´s HLA class I molecules, play a key role in the therapeutic efficacy of hematopoietic stem cell transplantation (HSCT). Alternatively, NK cells may be expanded, activated, or redirected against cancer cells (chimeric antigen receptor (CAR)-NK cells) ex vivo and adoptively transferred to patients with hematological cancers. The antitumor activity of NK cells may also be stimulated by cytokines or immunostimulatory drugs such as lenalidomide or pomalidomide. Due to their capability of stimulating NK cell activity and ADCC, they may have synergistic effects with therapeutic mAbs. Blocking antibodies directed against inhibitory NK cell receptors, including inhibitory KIRs (iKIR) (lirilumab) or natural killer group 2A (NKG2A) (monalizumab), and checkpoint proteins, including programmed death-1 (PD-1), have great clinical potential in this type of malignancies. Similarly, agonistic antibodies targeting T and NK cell costimulatory molecules, such as cluster of differentiation 137 (CD137)/4-1BB, are novel therapeutic alternatives for cancer therapy. HSC, hematopoietic stem cell; IMiDs, immunomodulatory drugs; mAbs, monoclonal antibodies. SLAMF7, SLAM family member 7.

Several current therapeutic strategies may restore or potentiate the ability of NK cells to eliminate cancer cells in hematological malignancies (Figure 2, and Table 2). These strategies include the following: (1) Therapeutic approaches that engage NK cell activating receptors are the most widely used in the clinic, particularly, mAbs that engage CD16 receptor on NK cells and induce ADCC activity. (2) HSCT is another key therapeutic strategy that harnesses the alloreactivity of NK cells. This strategy may be refined by the direct adoptive transfer of NK cells that may be previously expanded, activated, or redirected against cancer cells. (3) The activity of NK cells may also be boosted by cytokines and immunostimulatory drugs. (4) Finally, targeting inhibitory receptors and other immunosubversive mechanisms developed by hematological cancers may release the antitumor potential of NK cells, particularly, mAbs blocking NK cell inhibitory receptors and checkpoint proteins are novel promising therapeutic drugs in hematological cancers.

## 4. NK Cells Cell-Mediated ADCC

### 4.1. Anti-CD20 Antibodies

The use of tumor-specific mAbs that promote ADCC through the ligation of CD16 receptor on NK cells has become standard for many hematologic malignances (Table 3). CD16a/FcγRIIIA is expressed in the majority of NK cells and it is the only activating receptor with the capability of triggering alone, and even in the presence of inhibitory signals, the cytotoxic activity of NK cells [146]. IgG_1_ and IgG_3_ exhibit high affinity for CD16a, which directly correlates with their ability to trigger NK cells cell-mediated ADCC [147]. However, these antibodies have additional mechanisms of action including direct signaling and complement-dependent cytotoxicity (CDC); and the contribution of these mechanisms of action to the clinical efficacy is not fully understood [148]. The complete elucidation of the mechanism of action of these mAbs is essential to improve their therapeutic success and to overcome clinical resistance [149].

Rituximab is a mouse-human chimeric IgG_1_ mAb that targets CD20 antigen, which is displayed by B cells in a variety of differentiation stages and by most B cell-derived tumors, including more than 90% of B cell NHL and, to a lesser extent, in CLL. Remarkably, healthy B cells are also eliminated by rituximab causing a lymphopenia, but this is a transient effect typically lasting about 6 months [150]. Rituximab is now fully integrated into the management of patients with NHL or CLL, with most of them receiving rituximab as either a single agent or, more frequently, in combination with chemotherapy to improve efficacy [63,64,65]. Despite of the widespread use of rituximab, most mechanistic data have been obtained from in vitro and preclinical studies, while the role of the distinct effector mechanisms contributing to their therapeutic efficacy in humans are not completely elucidated and may vary depending on the malignancy [148]. Direct antineoplastic effect, ADCC and CDC, all appear to play a role in rituximab efficacy [66]. The most convincing evidence of the relevant role of ADCC in the efficacy of rituximab comes from the significant correlation between functional polymorphisms of the gene encoding CD16a/FcγRIIIa and clinical response to rituximab. Polymorphisms conferring higher affinity to IgG_1_ have better clinical response to rituximab in follicular lymphoma [67,68], Waldestrom´s macroglobulinemia [69], and patients with DLBCL treated with rituximab plus CHOP (R-CHOP) [70], however, CD16a/FcγRIIIa polymorphisms do not predict clinical response in CLL [71] and patients with follicular lymphoma treated with R-CHOP [72]. This evidence together with the fact that the therapeutic effect of rituximab was effective in wild-type mice, but not in mice lacking the common FcγR chain [73], strongly support that ADCC plays a central role in the clinical activity of rituximab. This has led to the development and clinical evaluation of novel therapeutic strategies for enhancing cancer cell elimination by NK cells including the addition to rituximab of immunostimulatory agents designed to activate NK cells [108,109,110], and the development of anti-CD20 antibodies with stronger affinity for CD16a [151] (Table 3). These strategies are under clinical evaluation and they will help us to understand the clinical importance of ADCC in the therapeutic efficacy of these mAbs. Additionally, this new generation anti-CD20 mAbs has been humanized [152] since rituximab retains a high antigenic potential to the human immune system due to the presence of murine immunoglobulin sequence, and therefore carries a risk of hypersensitivity reactions upon parenteral administration (infusion-related reactions) which may limit its efficacy.

Obinutuzumab is a fully humanized IgG_1_ anti-CD20 mAb recognizing a unique CD20 epitope [74]. Obinutuzumab has been glycol-engineered to reduce the amount of fucose attached to the antibody conferring increased affinity for CD16a leading to an increased ADCC activity in vitro [151]. Additionally, it has a modified elbow-hinge region resulting in enhanced direct cell death compared with rituximab and ofatumumab [151,153,154]. In agreement, obinutuzumab showed superior efficacy compared with rituximab in xenografts models [155,156,157,158] and it killed leukemia cells to a similar extent, but more rapidly than rituximab, in whole blood assays of B cell NHL and CLL patients [154]. Clinically, the ADCC function of rituximab and obinutuzumab is variable and their contribution to efficacy of these drugs is difficult to quantify. Nevertheless, the number and functionality of NK cells in treated patients significantly correlate with the success of anti-CD20-based therapies [159], which highlights a significant role of NK cells in the efficacy of these anti-CD20 mAbs. Clinical data are still preliminary and based on higher doses of obinutuzumab as compared with standard doses of rituximab, but obinutuzumab has shown an improved efficacy in the treatment of CLL and indolent lymphomas [66,160]. Whether these improvements are due to higher doses of obinutuzumab, or due to enhanced ADCC activity warrant further investigation [75]. Additional trials are also required to fully define the role of this new antibody in aggressive lymphomas [160].

**Table 3:** Monoclonal antibodies, which are currently approved for hematological malignancies, that promote NK cells cell-mediated ADCC. These mAbs have a direct antineoplastic effect and additional mechanisms of action including ADCC. The ADCC activity of these antibodies may vary among diseases and its relative contribution of these drugs to the efficacy remains to be fully established. ADCC, antibody-dependent cell-mediated cytotoxicity; R, rituximab; DLBCL, diffuse large-B-cell lymphoma; CLL, chronic lymphocytic leukemia; MM, multiple myeloma; CHOP, cyclophosphamide, doxorubicin, vincristine, prednisone; FC, fludarabine-cyclophosphamide; PFS, progression free survival; OS, overall survival, CR, complete remission; ORR, overall response rate.

mAb	Target	Trial	Disease	Intervention	Outcomes
**Rituximab (R)**	CD20	LNH98-5 Phase III [63]	DLBCL (age 60–80 years)	R-CHOP vs. CHOP	10 years; PFS: 37 vs. 20% 10 years; OS: 44 vs. 28%
		Mint Phase III [64]	DLBCL (younger with good prognosis)	R-CHOP vs. CHOP	6 years; PFS: 74 vs. 55%
		CLL8 trial Phase III [65]	Untreated CLL	R-FC vs. FC	CR 44% vs. 22% PFS: 57 vs. 33 months OS not reached vs. 86 months
**Obinutuzumab**	CD20	Gadolin Phase III [161]	Rituximab refractory indolent lymphoma	Obinutuzumab -Bendamustine vs. Bendamustine	PFS: 25 vs. 14 months OS: not reached vs. 54 months
		Gallium Phase III [162]	Advanced stage follicular lymphoma	Obinutuzumab-chemotherapy vs. Rituximab- chemotherapy	3 years; PFS: 80% vs. 73%
		CLL11 Phase III [163]	CLL (elderly with comorbidities)	Obinutuzumab- chlorambucil vs. R- chlorambucil vs. chlorambucil	PFS months: 27 (Ob-Cl), 16 (R-Cl), 11 (Cl)
**Daratumumab**	CD38	Castor Phase III [164]	Relapsed/ refractory MM	Daratumumab- Bortezomib- dexamethasone vs. Bortezomib- dexamethasone	PFS: 16.7 vs. 7.1 months ORR: 83% vs. 63%
		Pollux Phase III [165]	Relapsed/ refractory MM	Daratumumab- lenalidomide- dexamethasone vs. lenalidomide- dexamethasone	PFS not reached vs. 17.5 months ORR: 92% vs. 76%
**Elotuzumab**	SLAMF7 (CS1)	Eloquent Phase III [166]	Relapsed/ refractory MM	Elotuzumab- lenalidomide- dexamethasone vs. lenalidomide- dexamethasone	4 years; PFS: 21% vs. 14% ORR: 79% vs. 66%

Ofatumumab is another novel anti-CD20 mAb that binds to a distinct site at the CD20 transmembrane protein, and it is more efficient recruiting C1q and activating the classical pathway of complement [167]. It remains to be elucidated whether this increase of CDC activity is clinically relevant. Preclinical data showed stronger antitumor effect of obinutuzumab as compared to ofatumumab [157]. Head-to-head randomized clinical trials comparing these new anti-CD20 mAbs are needed to draw definite conclusions. This will be further complicated with the development of new anti-CD20 antibodies with enhanced properties that are currently in progress.

### 4.2. Anti-CD38 Antibodies

Daratumumab and elotuzumab are cytotoxic mAbs approved for the treatment of MM (Table 3). Daratumumab is a fully humanized IgG_1_ mAb targeting CD38, a transmembrane glycoprotein that is overexpressed on myeloma cells. CDC was initially described as the mechanism of action of daratumumab. Additionally, several other mechanisms of action have been recently described including ADCC [76,77]. Nevertheless, the relative contribution of NK cells cell-mediated ADCC on the clinical efficacy of daratumumab has not been well established. Furthermore, CD38 is also expressed on other immune cells including NK cells. Thus, treatment with daratumumab may reduce the number of NK cells, although the NK cells are not completely depleted, and they may still contribute to ADCC and clinical efficacy [78]. Additionally, the tumor microenvironment protects myeloma cells from CD38 antibody induced ADCC by upregulating anti-apoptotic proteins, such as survivin [79].

### 4.3. Elotuzumab

Elotuzumab is a humanized IgG_1_ mAb that induces NK cell activation by binding to SLAM family member 7 (SLAMF7) (also known as CS1), which is highly expressed on NK cells, plasma cells, and myeloma cells (>95%) [80]. The mechanism of action of elotuzumab in MM involves the activation of NK cells through CD16-mediated ADCC [81]. Interestingly, elotuzumab does not exert a cytotoxic activity against NK cells, instead, it acts as an agonist antibody further contributing to NK cell activation.

### 4.4. Novel Antibodies

Multiple antibodies targeting hematological malignancies are being evaluated in preclinical models or clinical trials. Bispecific antibodies (BiKEs) are likely the most promising of those antibodies. BiKEs are engineered to join two antigen-binding domains of two antibodies in a unique molecule targeting several epitopes simultaneously. Initial bispecific T cell engagers (BITEs) were developed to redirect T cell-mediated cytotoxic activity against B cell-derived cancer cells [82]. More recently, a new generation of NK cell-stimulating BiKEs or trispecific antibodies (TRiKEs) are being developed. They activate NK cells against one or more tumor antigens through CD16a. Overall, few clinical data are still available since bispecific NK cell engagers have only recently entered into the clinic. Nevertheless, they show a significant efficacy and superior safety profiles in initial clinical or late stage preclinical development [83]. Interestingly, they have been shown to increase NK cell-mediated cytotoxicity and cytokine production in leukemias and lymphomas [84]. A bispecific antibody targeting CS1-NKG2D has shown to prolong survival in a MM model [85].

## 5. Hematopoietic Stem Cell Transplantation and Adoptive Transfer of NK Cells

HSCT offers the only chance of cure for many hematological malignancies. Its therapeutic effect is mainly mediated by the immune response against cancer cells exerted by the donor´s T and NK cells in a process known as graft-versus-leukemia (GvL) effect [86]. A major complication of HSCT is graft-versus-host disease (GvHD) that is caused by allogenic T lymphocytes. Conversely, NK cells recover quickly after HSCT and do not cause GvHD and they may even protect against GvHD by targeting the recipient’s dendritic cells [54,55,87]. Consequently, NK cells represent an attractive target to improve the anti-leukemia properties after HSCT without increasing its toxicity.

Allogenic NK cells can eliminate a recipient´s cancer cells if they express low levels of HLA-I molecules or express HLA-I alleles that are not recognized by inhibitory KIRs of donor NK cells. The latter is owing to the fact that KIR genes are polygenic and polymorphic, with different numbers of activating and inhibitory KIR genes found in different individuals. Six inhibitory receptors recognize epitopes shared by certain allelic variants of HLA-I molecules. Particularly relevant are KIR2DL1 and KIR2DL2/3, which recognize some HLA-C alleles and KIR3DL1 specific for some HLA-B and HLA-A alleles. To establish self-tolerance, the inhibitory KIRs and HLA-I alleles of each individual structure the NK cell repertoire during development. Thus, engagement of inhibitory KIR with self HLA-I molecules educates, or “licenses”, NK cells for function [88]. As KIR genes (chromosome 19q13.4) and HLA genes (chromosome 6p21) segregate independently, only about 25% of the HLA-matched sibling donor-recipient pair share identical KIRs. A seminal study in T cell depleted haploidentical transplants in AML showed that patients lacking KIR ligands (HLA-I alleles) present in haploidentical donors (“missing self”) experienced reduced risk of leukemia relapse (75% vs. 0% at 5 years), did not cause GvHD and increased overall survival [54,55]. This study shows how unleashing the potential of NK cells may translate into outstanding clinical responses in hematological malignancies. Nevertheless, the beneficial effect of KIR-HLA mismatch was mainly limited to patients with AML for unknown reasons [89]. It should be also noted that some studies have even identified an adverse effect of KIR-ligand mismatch on overall survival in haploidentical transplants and cord-blood transplants [90,91]. Difference in graft source (e.g., peripheral blood and bone marrow), graft processing (T cell depletion), and GvHD prophylaxis may significantly influence NK cell biology. Additional studies will be needed to establish the most adequate conditions to boost NK cell activity in these patients.

The function of activating KIRs remains an open question, but specific KIR activating genes, such as KIR3DS1, have been associated with less GvHD in allogeneic HSCT [92], whereas others, such as KIR2DS1, protect from leukemia relapse [93,94]. Two main haplotypes encoding KIR genes, A and B that correlate with a more inactivating or activating phenotype, have been reported. The A haplotypes are mainly composed by inhibitory receptors and just one activating KIR (2DS4), whereas B haplotypes are formed by one or more activating receptors (*KIR2DS1/2/3/5*, *KIR3DS1*) [95]. There is an emerging consensus that donor KIR B haplotypes, which contain a higher content of activating KIR genes, are more beneficial for unrelated donor transplant outcome in leukemia [94], lymphoma [96], and also in related donors [97].

### 5.1. Adoptive Transfer of NK Cells

Compared to HSCT, one step further is the direct adoptive transfer of NK cells to cancer patients. Autologous or allogenic NK cells, NK cell lines, or chimeric antigen receptor (CAR) NK cells may be used as a source for adoptive transfer. Autologous NK cells are activated and expanded ex vivo and reinfused to relapsed MM patients. Despite being a safe procedure, clinical responses are limited [98]. Ongoing studies are being developed to increase the activity and persistence of infused NK cells. For instance, NK cells isolated from related HLA-haploidentical donors were activated with the CTV-1 leukemia cell line lysate CNDO-109 resulting in enhanced cytotoxicity and NK cell activation in high-risk patients with AML in a phase I clinical trial [99]. Alternatively, NK cells may be pre-activated with interleukin (IL)-12, IL-15, and IL-18 improving clinical responses in patients with AML [100]. Despite some initial promising results, further studies and additional strategies are clearly warranted before this approach can enter into the clinic.

### 5.2. CAR-NK Cells

Genetic engineering may also be used to increase the effectiveness of adoptively transferred NK cells. CAR-T cells have obtained impressive clinical results in ALL and NHL, thus, becoming a major breakthrough in cancer therapy [101]. Theoretically, NK cells may be an alternative driver for CARs. CAR-NK cells are considered safer than CAR-T cells because they are short living cells and produce cytokines, including IFN-γ and GM-CSF, with a lower toxicity profile than those produced by T cells [102]. Additionally, CAR-NK cells are equipped with an innate cytotoxic activity provided by an array of activating receptors, which may potentiate the activity of the CAR. Particularly, the expression of CD16 may endow CAR-NK cells with ADCC activity, providing a rationale support for combining this therapy with mAbs. An important drawback of the use of NK cells is the fact that they are difficult to obtain and manipulate [103]. CAR-NK cells targeting several different antigens, including CD19, CD20, CD33, CD138, SLAMF7, CD3, CD5, and CD123, using both primary NK cells and the NK cell line NK-92, are currently being investigated in preclinical and initial clinical trials [104]. A recent phase I clinical trial using CD33-directed CAR-NK-92 cells in patients with relapsed refractory AML showed no major adverse effects, indicating that CAR-NK cells may be a safe alternative for CAR-T cells [105]. CD19-directed CAR-NK-92 also showed increased cytotoxic activity against CD19-leukemia cells [106]. Nevertheless, CAR-NK cells show limited persistence, low capacity of infiltrating tumor sites, and reduced cytotoxicity in vivo. To improve the proliferation and persistence of NK cells, an optimized CAR-NK cell construct including the coding sequence of IL-15 has been developed and it is currently being evaluated (NCT03056339). Of interest, CD20-targeting NK-92 cells have showed an increased antitumor activity against primary CLL cells as compared with rituximab and ofatumumab in vitro, indicating that CAR-NK cells could be an efficient alternative to cytotoxic mAbs [107].

## 6. Cytokine Therapy and Immunomodulatory Drugs

Another key therapeutic strategy to increase the antitumor activity of NK cells exists in their stimulation with cytokines and immunomodulatory drugs (IMiDs). Cytokines have been considered as promising agents for cancer treatment for long time. Nevertheless, their pleiotropism and redundancy have dampened their clinical use. Certain T cell- and NK cell-stimulating cytokines, such as IL-2, have gained notoriety in the treatment of some solid tumors, particularly in metastatic melanoma and metastatic renal cancer [13]. Similarly, certain interferons that stimulate NK cell activity, such as interferon-α, have been used; nevertheless, IMiDs have become the most relevant immunomodulatory drugs in hematological cancers.

### Immunomodulatory Drugs

Lenalidomide is an orally active IMiD with significant activity in MDS, MM, and NHL. Lenalidomide has a pleotropic mechanism of action with direct antineoplastic activity and indirect effects including inhibition of angiogenesis and immunomodulation of multiple cell types present in a tumor microenvironment, such as B, T, NK, and dendritic cells. The relative contribution of the different mechanisms of action of lenalidomide has not been well established, but the immunomodulation and, particularly, the activation of NK cells is increasingly recognized as important in tumor recognition and elimination [110,111].

At the molecular level, lenalidomide binds and activates cereblon, a ubiquitously expressed ubiquitin E3 ligase [112], resulting in rapid ubiquitination and degradation of multiple substrates, including Ikaros and Aiolos transcription factors, which are involved in the development and function of lymphocytes including T cells [113]. A key consequence of lenalidomide treatment in T cells is the enhancement of IL-2 production, a key cytokine involved in the activation and proliferation of both T and NK cells [114]. Lenalidomide exerts its effect on NK cells by both direct (via cereblon) and indirect mechanisms, which may vary with the cancer type. For instance, we showed that lenalidomide markedly increased the activation and proliferation of CD4 T cells and NK cells in CLL [115,116], as its effect on NK cells was mainly due to the induction of IL-2 production by CD4 T cells [115,116]. Lenalidomide treatment increases the activation and number of NK cells, promotes the expression of activating receptors, such as CD16 [117], and enhances the NK cell natural cytotoxicity and ADCC [118]. Lenalidomide also decreases the expression of NK cell inhibitory receptors, such as Ig-like transcript 2 (ILT2), which is profoundly dysregulated in CLL [119,120], and decreases both the expression of both PD-1 on NK cells and its ligand, programmed death-ligand 1 (PD-L1), on tumor cells [117]. Clinically, lenalidomide promotes NK cell expansion and enhances NK cell activity achieving clinical responses in relapsed-refractory CLL, follicular lymphoma, small lymphocytic lymphoma, mantle cell lymphoma, and DLBCL [108,109,110].

Pomalidomide is an analogue of lenalidomide with a potent immunomodulatory activity that leads to a rapid decline in the transcription factor Ikaros in T and NK cells stimulating their antitumor activity, even in heavily pretreated patients with MM, suggesting that pomalidomide shares a similar mechanism of action with lenalidomide [124,125].

The mechanism of action of lenalidomide provides a rationale support for its combination with mAbs. Combining lenalidomide with rituximab resulted in an increase of ADCC against various NHL cell lines [121] and in reactivation of dysfunctional NK cells in vivo, leading to increased cytokine production and immune synapse signaling [110]. Lenalidomide in combination with obinutuzumab induced the activation of circulating NK cells and reversed their immature phenotype [122]. Lenalidomide was also shown to upregulate CD38 surface expression on myeloma cells, priming them for daratumumab-induced NK cell-mediated ADCC [123]. Large phase III clinical trials with lenalidomide in combination with cytotoxic ADCC-inducing mAbs have shown significantly improved response rates and survival in NHL and MM patients (source: https://clinicaltrials.gov/). Elucidating the role of NK cells in the clinical efficacy of these new therapies may be important to develop new and more efficient treatments for these patients.

## 7. Targeting Inhibitory Receptors and Checkpoint Proteins

Blocking inhibitory pathways dampening NK cell function in cancer is a novel and promising strategy for immunotherapy [126]. Anti-PD-1/PD-L1 blockade has shown impressive clinical results in patients with melanoma, causing durable tumor regression in a significant group of patients [127]. With the exception of the anti-PD-L1 antibody avelumab [128], these mAbs do not induce ADCC, instead, the clinical benefit of these therapies is thought to mainly rely on the reactivation of exhausted T cells. Nevertheless, exhausted NK cells also express PD-1 and it has been shown that NK cells also play a significant role in the efficacy of anti-PD-1 therapy in experimental models and in patients with melanoma [129]. Similarly, PD-1 is overexpressed on NK cells in a variety of hematological malignancies, and accumulating evidences suggest that NK cells are also important players in the efficacy of anti-PD-1/PD-L1 therapy in this type of malignancies [117,130,131,132,133,134,135]. They are particularly relevant in HL since PD-L1 and PD-L2 are overexpressed in the vast majority of patients due to the amplification of 9p24.1 locus containing both genes [136]. Development of anti-PD-1 blocking-based therapies, alone or in combination, is currently being explored in several clinical trials (source: https://clinicaltrials.gov/). The potential role of NK cells in the efficacy of these mAbs remains to be elucidated.

Other mAbs targeting NK cell inhibitory receptors are being explored. Lirilumab is a pan-KIR2D blocking antibody for inhibitory KIRs (KIR2DL/DS-1, -2, and -3) that prevents KIR-HLA-C interaction. KIR-blocking antibodies might mimic the missing-self scenario, stimulating NK cell-mediated responses. Despite the fact that preclinical and initial clinical data were promising, lirilumab monotherapy failed to show effectivity in patients with AML and MM, probably due to the reduced expression of KIRD2 and the lack of NK cell responsiveness [137].

CD94-NKG2A is another inhibitory receptor that binds to HLA-E, a nonclassical HLA-I molecule that mediates self-tolerance. HLA-E overexpression is a widely used mechanism of cancer immune evasion, particularly in hematological malignancies [138,139,140,141]. Monalizumab is a humanized IgG_4_ mAb that blocks NKG2A-HLA-E interaction. Preclinical studies have demonstrated a potent antitumor activity of monalizumab in hematological cancers [139,142], and several clinical trials that have used monalizumab as monotherapy or in combination are currently ongoing. Of note, NKG2A and HLA-E play a central role in NK cell-mediated immune evasion after allo-HSCT [138,143], and monalizumab may significantly enhance the NK cell-mediated GvL effect [142].

Multiple inhibitory receptors are overexpressed and play a key role in the modulation of the immune response in hematological malignancies [126]. Tim-3, lymphocyte activation gene 3 (LAG-3), T cell Ig and ITIM domain (TIGIT), and CD96 (also known as TACTILE) are inhibitory receptors that may become relevant therapeutic targets in the near future.

Contrasting with the blockade of inhibitory receptors, agonistic mAbs targeting T and NK cells costimulatory molecules, such as cluster of differentiation 137 (CD137/4-1BB), significantly inducing NK cell activity [144,145]. Two agonist CD137 mAbs, Urelumab (human IgG_4_ mAb) and Utomilumab (humanized IgG_2_), are currently being tested in hematological malignancies.

## 8. Conclusions

NK cells have the innate ability to kill cancer cells in vitro, however, cancer cells are able to evade the immune system leading to malignancies. Restoring or potentiating this natural antitumor activity of NK cells has become a key therapeutic approach in cancer and, particularly, in hematological cancers. NK cells are a universal source of tumor-killer cells for cancer therapy since autologous, heterologous, or even NK cell lines may be used. The antitumor activity of NK cells may be directly boosted in patients with cancer or previously manipulated, activated, or redirected ex vivo. Several approaches that potentiate the antitumor activity of NK cells have been developed. Some of them have been successfully used for decades such as mAbs or HSCT. These successful therapies have laid the foundation for the development of new NK cell-based cancer therapies. Some of these novel therapies, including CAR-NK cells, BiKEs, and TRiKEs, are very promising, whereas others, such as the adoptive transfer of NK cells, have obtained discouraging results. Nevertheless, the recent advance in the understanding of the biology of NK cells along with the improvement in the field of cell engineering, genetics, and proteomics may significantly increase the range of patients who will be treated with NK cell-based therapy in a near future.

## Figures and Tables

**Table 2:** Current and most promising NK cell immunotherapies used in hematological cancers. ADCC, antibody-dependent cell-mediated cytotoxicity; CDC, complement-dependent cytotoxicity; HSCT, hematopoietic stem cell transplantation; CAR, chimeric antigen receptor CLL, chronic lymphocytic leukemia; AML, acute myeloid leukemia; GvHD, graft-versus host disease.

Therapy	Features	Disadvantages	References
**NK Cells Cell-Mediated ADCC**			
Rituximab	Clinical benefits in most B-cell lymphomas and CLL	Risk for hypersensitivity reactions	[63,64,65,66,67,68,69,70,71,72,73]
Obinutuzumab	Increases ADCC	Limited clinical experience	[67,68,69,70,71,72,73]
Ofatumumab	Increases CDC	Clinical relevance not established	[74,75]
Daratumumab	Induces ADCC and CDC	Role of NK cells not elucidated	[76,77,78,79]
Elotuzumab	Induces NK cell activity and ADCC	Anti-elotuzumab antibodies may limit efficacy	[80,81]
BiKEs/TRiKEs	Redirect NK cells	Limited clinical experience	[82,83,84,85]
**HSCT and Adoptive Transfer**			
HSCT	Highly efficient in KIR-HLA mismatched patients. No risk of graft vs host disease (GvHD)	Efficacy only demonstrated in AML	[54,55,86,87,88,89,90,91,92,93,94,95,96,97]
Autologous/ Allogenic NK cells	Universal use. No risk of GvHD	Low persistence, activity and efficacy of NK cells	[98,99,100]
CAR-NK cells	Potent antitumor activity and safer than CAR-T cells	Difficult to manipulate, limited persistence and efficacy	[101,102,103,104,105,106,107]
**Immunomodulatory Drugs**			
Lenalidomide	Induces NK cell activity and ADCC	Combination with dexamethasone may affect NK cells	[108,109,110,111,112,113,114,115,116,117,118,119,120,121,122,123]
Pomalidomide	Increases effectiveness	May induce inhibitory checkpoints expression on NK cells	[124,125]
**Inhibitory Receptors**			
PD-1 and PD-L1	Relevant results in patients with HL	Role of NK cells not elucidated	[126,127,128,129,130,131,132,133,134,135,136]
Lirilumab	Mimics missing-self scenario	Low clinical efficacy, needs combination	[137]
Monalizumab	Induces NK cell activity. Potential for combination with HSCT in AML	No clinical data available	[138,139,140,141,142,143]
Urelumab and Utomilumab	Induces NK cell activity and ADCC	High toxicity and/or low clinical efficacy as monotherapy	[144,145]
